# Common Spatio-Time-Frequency Patterns for Motor Imagery-Based Brain Machine Interfaces

**DOI:** 10.1155/2013/537218

**Published:** 2013-11-03

**Authors:** Hiroshi Higashi, Toshihisa Tanaka

**Affiliations:** ^1^Department of Electronic and Information Engineering, Tokyo University of Agriculture and Technology, 2-24-16 Nakacho, Koganei-shi, Tokyo 184-8588, Japan; ^2^RIKEN Brain Science Institute, 2-1 Hirosawa, Wako-shi, Saitama 351-0106, Japan

## Abstract

For efficient decoding of brain activities in analyzing brain function with an application to brain machine interfacing (BMI), we address a problem of how to determine spatial weights (spatial patterns), bandpass filters (frequency patterns), and time windows (time patterns) by utilizing electroencephalogram (EEG) recordings. To find these parameters, we develop a data-driven criterion that is a natural extension of the so-called common spatial patterns (CSP) that are known to be effective features in BMI. We show that the proposed criterion can be optimized by an alternating procedure to achieve fast convergence. Experiments demonstrate that the proposed method can effectively extract discriminative features for a motor imagery-based BMI.

## 1. Introduction

 Brain machine/computer interfacing (BMI/BCI) is a challenging technology of signal processing, machine learning, and neuroscience [1]. BMIs capture brain activities associated with mental tasks and external stimuli, realize nonmuscular communication, and control channel for conveying messages and commands to the external world [1–3]. Basically, noninvasively measured data such as electroencephalogram (EEG), magnetoencephalogram (MEG), and functional magnetic resonance imaging (fMRI) are widely used to observe brain activities. Among them, because of its simplicity and low cost, EEG is practical for use in engineering applications [4, 5].

Efficient decoding around motor-cortex is a crucial technique for the realization of BMI associated with motor-imagery (MI-BMI) [6, 7] with the application to controlling external devices [7], prostheses [4], rehabilitation [8], and so forth. For instance, it is also known that the real and imaginary movements of hands and feet evoke the change of the so-called mu rhythm in different brain regions [2, 3]. Therefore, the accurate extraction of these changes from the measured EEG signals in the presence of measurement noise and spontaneous components which are related to other brain activities enables us to classify the EEG signal associated with the different motor (imagined) actions such as movement of the right hand, left hand, or feet.

In classification of EEG signals in MI-BMI and analyzing of the brain activities during motor imagery, signal processing techniques such as bandpass filtering and spatial weighting are used [1]. For the processing, presuming the parameters such as coefficients of the filters and weights that extract the related components is a crucial issue. Moreover, the optimum parameters in classification are highly dependent on users and measurement environments [9].

In order to determine the parameters, data-driven techniques that exploit observed data are widely used [1, 2]. The observed data essentially include class labels corresponding to the tasks. The techniques should find the parameters that extract discriminative features as much as possible. For example, the well-known common spatial pattern (CSP) method finds the spatial weights by using the observed signals [1, 9, 10] in such a way that the variances of the signals extracted by the linear combination of a multichannel signal and the spatial weights differ as much as possible between two classes. The standard CSP method has been extended to methods to estimate the other parameters, such as the frequency bands [11–16], and methods to select the CSP features extracted with various parameters [17, 18].

Besides, one of the parameters to be decided by data-driven techniques is a time window because of the following reasons. A kind of BMIs is implemented based on cues which a user follows. In the BMI, the user begins to perform a task when the cue is given. Therefore, the time when the user begins to perform the task is known. However, the time when the brain activity associated with the task occurs is unknown. The time windows working to remove samples that do not contain the brain activity will not match the period when the cues are showed. For instance, the samples for a few hundreds of milliseconds after the cues are assumed not to be used to extract the features in previous works [13, 17, 19, 20], which heuristically determined the time window. Contrary to these works, this paper hypothesizes that an optimal observation period in classification depends on users. For example, reaction time defined as the elapsed time between the presentation of a sensory stimulus and the subsequent behavioral response is strongly associated with age [21]. The reaction time can be related to the time of response to the cues. Therefore, the time window should also be designed by using the observed signals or data-driven.

In this paper, we propose a method for finding the time windows as well as the aforementioned parameters (CSP and temporal filters) by extending a framework proposed in [15]. The proposed method enables us to find these parameters that separately extract several components that are observed in different spatial patterns, frequency bands, and periods of time. We call these simultaneously designed parameters common spatio-time-frequency patterns (CSTFP). As compared to the methods, in which the parameters are selected out of the predefined candidates fixed in advance [18], the CSTFP method has a higher degree of freedom for the parameters reducing the computational costs. In CSTFP, the coefficients of the temporal filters and the spatial weights are searched in the set of real numbers. Moreover, although the time windows are selected out of a set of candidates, the computational cost of the CSTFP method does not grow rapidly even with a large number of candidates.

The rest of this paper is organized as follows.  Section 2.1 reviews the CSP method. Next we, illustrate the CSTFP method from Section 2.2 to Section 2.4. We experimentally analyze the CSTFP method by using artificial data in Section 3.  Section 4 presents experimental results of classification of EEG signals during motor imageries to show the performance of CSTFP. Finally, the conclusions of this paper are presented in Section 5.

## 2. Common Spatio-Time-Frequency Patterns (CSTFP)

We describe a novel method called the CSTFP method to simultaneously find the parameters for the spatial weights, the temporal filters, and the time windows. In this section, before introducing CSTFP, CSP is reviewed. Next, we define a signal extraction model using the spatial weighting, filtering, and time windowing. Then, we propose a criterion designing the parameters motivated by that for the CSP method. The proposed criterion evaluates not only the spatial weights but also the coefficients of the time windows and the temporal filters. Next, the optimization method for the proposed criterion with alternate updating is presented. Finally, we define feature vector with the feature extraction model for classification of unlabeled EEG signals.

### 2.1. Common Spatial Pattern (CSP): A Review

Let **X** ∈ ℝ^*M*×*N*^ be an observed signal, where *M* is the number of channels and *N* is the number of samples. In BMI application, we do not directly use **X**, but we use the filtered signal described as $\hat{X}=ℋ(X)$ to find the CSP, where *ℋ* is a bandpass filter which passes the frequency components related to brain activity of motor imagery. Denote the components of $\hat{X}$ by $\hat{X}=[{\hat{x}}_{1},\ldots ,{\hat{x}}_{N}]$, where ${\hat{x}}_{n}\in {\mathbb{R}}^{M}$ and *n* is the time index. We assume the sets of the observed signals, *𝒞*_1_ and *𝒞*_2_, where *𝒞*_*d*_ contains the signals belonging to class *d*, *d* ∈ {1,2} is a class label, *𝒞*_1_∩*𝒞*_2_ = *∅*, and *∅* is a set having no elements. CSP is defined as the weight vector that maximizes the intra class variance in *𝒞*_*c*_ under the normalization of samples, where *c* is a class label. More specifically, for *c* being fixed, the weight vector is found by solving the following optimization problem [9, 10]:

(1) max⁡w EX∈𝒞c[1N∑n=1N|wT(x^n−μ)|2],subject  to ∑d=1,2EX∈𝒞d[1N∑n=1N|wT(x^n−μ)|2]=1,

where *E*_**X**∈*𝒞*_*d*__[·] denotes the expectation over *𝒞*_*d*_,  ***μ*** is the time average of **X** given by $\mu =(1/N)\sum_{n=1}^{N}{\hat{x}}_{n}$, ·^*T*^ is the transpose of a vector or a matrix, and |·| is the absolute value of a scalar. The solution of (1) is given by the generalized eigenvector corresponding to the largest generalized eigenvalue of the generalized eigenvalue problem described as

(2) $$\begin{array}{c} \Sigma_{c}w=\lambda \left(\Sigma_{1}+\Sigma_{2}\right)w, \end{array}$$

where Σ_*d*_, for *d* = 1,2, are defined as $\Sigma_{d}=E_{X\in {𝒞}_{d}}\left[(1/N)\sum_{n=1}^{N}\left({\hat{x}}_{n}-\mu \right){\left({\hat{x}}_{n}-\mu \right)}^{T}\right]$.

### 2.2. Signal Extraction Model

**Figure 1:**
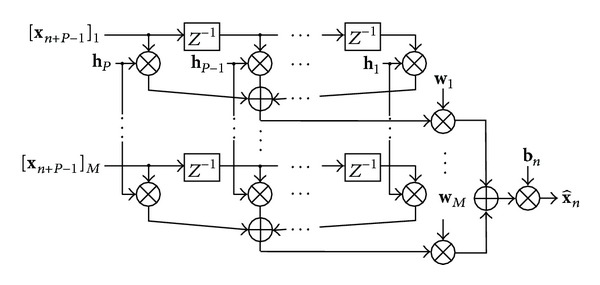
The filtering of an observed signal by a temporal filter, **h**, spatial weights, **w**, and a time window, **b**. *Z* ^−*n*^ is an operator of *n* samples delay.

 The target signal and signal extraction procedure are formulated in this section. The filtered signal of a target signal, **X**, denoted as $\hat{x}={\left[{\hat{x}}_{1},\ldots ,{\hat{x}}_{K}\right]}^{T}$, is defined as

(3) $$\begin{array}{c} {\hat{x}}_{n}=b_{n}\overset{M}{\underset{m=1}{\sum }}w_{m}\overset{P}{\underset{p=1}{\sum }}h_{p}{\left[x_{n+P-p}\right]}_{m}, \end{array}$$

for *n* = 1,…, *K*, *K* = *N* − *P* + 1, where [·]_*i*_ is the *i*th entry of a vector, *P* is the filter order of an FIR filter of which the coefficients are denoted by *h*_1_,…, *h*_*P*_, *w*_*m*_ is a spatial weight for *m*th channel, and *b*_*n*_ is a time window for *n*th sample that takes a binary value of either 0 or 1. The structure of temporally filtering, spatial weighting, and windowing for **X** is illustrated in Figure 1. In this model, **w**_*m*_ is regarded as the spatial pattern, **h**_*p*_ is regarded as the frequency pattern, and **b**_*n*_ is regarded as the time pattern of the extracted signal.

The sample variance of ${\hat{x}}_{n}$ over time *n* = 1,…, *K* is described as

(4) $$\begin{array}{c} \alpha_{X}\left(w,h,b\right)=\frac{1}{||b||}\overset{K}{\underset{n=1}{\sum }}{\left|{\hat{x}}_{n}-\frac{1}{K}\overset{K}{\underset{i=1}{\sum }}{\hat{x}}_{i}\right|}^{2}, \end{array}$$

where **w** is defined as **w** = [*w*_1_,…, *w*_*M*_]^*T*^, **h** is defined as **h** = [*h*_1_,…, *h*_*P*_]^*T*^, **b** is defined as **b** = [*b*_1_,…, *b*_*N*_]^*T*^, and ||·|| is the Euclidean norm of a vector.

The variance defined in (4) can be transformed to matrix-vector form as follows. We define **A**_*n*_, *n* = 1,…, *K*, whose elements are from **X** as

(5) $$\begin{array}{c} {\left[A_{n}\right]}_{m,p}={\left[X\right]}_{m,n+P-p}, \end{array}$$

for *m* = 1,…, *M* and *p* = 1,…, *P*, where [·]_*i*,*j*_ is the element at the *i*th row and the *j*th column of a matrix. Then, (4) can be modified to

(6) $$\begin{array}{c} \alpha_{X}\left(w,h,b\right)=\frac{1}{||b||}\overset{K}{\underset{n=1}{\sum }}b_{n}{\left|w^{T}{\hat{A}}_{n}h\right|}^{2}, \end{array}$$

where ${\hat{A}}_{n}$ is defined as ${\hat{A}}_{n}=A_{n}-{||b||}^{-1}\sum_{m=1}^{K}b_{m}A_{m}$.

### 2.3. Optimization for Sets of Parameters

We consider the problem of the design of *F* sets of the FIR filter, the spatial weights, and the time window represented by {**w**_i_, **h**_*i*_, **b**_*i*_}_*i*=1_^*F*^. These sets of the parameters are designed in such a way that **w**_*i*_, **h**_*i*_, and **b**_*i*_ maximize expectation of *α*_**X**_(**w**_*i*_, **h**_*i*_, **b**_*i*_) with respect to **X** ∈ *𝒞*_*c*_ samples under the normalization of an expectation of *α*_**X**_(**w**_*i*_, **h**_*i*_, **b**_*i*_) over all of the observation. Additionally, we impose the orthonormality on **h**_*i*_, *i* = 1,…, *F* to avoid the trivial solution. Moreover, the time windows are chosen from given candidates for efficient optimization. Therefore, we formulate the following maximization problem:

(7) max⁡𝒫i,i=1,…,F ∑i=1FJ^(𝒫i)+ϵK||bi||,subject  to hiThj||hi||||hj||=δij, i,j=1,…,F,hk∈𝒮k⊥, k=1,…,F,bl∈ℬ, l=1,…,F,

where *𝒫*_*i*_ represents set {**w**_*i*_, **h**_*i*_, **b**_*i*_} and $\hat{J}({𝒫}_{i})$ is the cost evaluating the ratio of the feature value in all samples defined as

(8) $$\begin{array}{c} \hat{J}\left({𝒫}_{i}\right)=\frac{E_{X\in {𝒞}_{c}}\left[\alpha_{X}\left(w_{i},h_{i},b_{i}\right)\right]}{\sum_{d=1,2}^{}E_{X\in {𝒞}_{d}}\left[\alpha_{X}\left(w_{i},h_{i},b_{i}\right)\right]}, \end{array}$$

where *𝒮*_*i*_ is any subspace in ℝ^*P*^, *ℬ* is a candidate set for the time windows, defined as *ℬ* = {**b**_*l*_}_*l*=1_^*L*^, *c* is a class label chosen from 1 and 2, *ϵ* is a regularization parameter, and *δ*_*ij*_ is the Kronecker delta defined as 1 for *i* = *j* and 0, otherwise.

Since it is difficult to simultaneously find all parameters, we consider sequential optimization to find the parameters with respect to each filter index *i*. That is, we first find *𝒫*_1_, and then find *𝒫*_2_ under the constraint on **h**_1_. This sequential optimization is represented with respect to each *i* as

(9) max⁡𝒫i   J^(𝒫i)+ϵK||bi||,subject  to   hi∈𝒮^i⊥, b∈ℬ,

where ${\hat{𝒮}}_{i}$ is a subspace defined as

(10) $$\begin{array}{c} {\hat{𝒮}}_{i}=\mathrm{Span}\left\{h_{1},\ldots ,h_{i-1}\right\}⊕{𝒮}_{i}\in {\mathbb{R}}^{P}, \end{array}$$

where *Span*⁡(⋯) represents a subspace spanned by vectors and the operator denoted by ⊕ gives the direct sum of two subspaces. Methods for choosing ${\hat{𝒮}}_{i}$ has been discussed in [15]. In (9), to optimize the parameters indexed with *i*, we adopt an alternating optimization procedure based on alternating least square (ALS). In the optimization, we separate the problem of (9) into three subproblems for **w**_*i*_, **h**_*i*_, and **b**_*i*_, respectively. Then, we update the parameters by alternating solving the subproblems. The three subproblems and these solutions are as follows.

The first subproblem is to optimize **w**_*i*_. While fixing **h**_*i*_ and **b**_*i*_, **w**_*i*_ maximizing (9) is found as the generalized eigenvector corresponding to the largest generalized eigenvalue of the generalized eigenvalue problem [15] described as

(11) $$\begin{array}{c} R_{c}w_{i}=\lambda \left(R_{1}+R_{2}\right)w_{i}, \end{array}$$

where

(12) $$\begin{array}{c} R_{d}=E_{X\in {𝒞}_{d}}\left[\frac{1}{||b_{i}||}\overset{K}{\underset{n=1}{\sum }}{\left[b\right]}_{n}{\hat{A}}_{n}h_{i}h_{i}^{T}{\hat{A}}_{n}^{\text{T}}\right], \end{array}$$

for *d* = 1,2, and *λ* is an eigenvalue.

The second subprolem is to optimize **h**_*i*_. While fixing **w**_*i*_ and **b**_*i*_, **h**_*i*_ maximizing (9) is found as the generalized eigenvector corresponding to the largest generalized eigenvalue of the generalized eigenvalue problem [15] described as

(13) $$\begin{array}{c} GQ_{c}h_{i}=\zeta \left(Q_{1}+Q_{2}\right)h_{i}, \end{array}$$

where

(14) $$\begin{array}{c} Q_{d}=E_{X\in {𝒞}_{d}}\left[\frac{1}{||b_{i}||}\overset{K}{\underset{n=1}{\sum }}{\left[b_{i}\right]}_{n}{\hat{A}}_{n}^{T}w_{i}w_{i}^{T}{\hat{A}}_{n}\right], \end{array}$$

for *d* = 1,2,

(15) $$\begin{array}{c} G=I_{P}-V{\left(V^{T}{\left(Q_{1}+Q_{2}\right)}^{-1}V\right)}^{-1}V^{T}{\left(Q_{1}+Q_{2}\right)}^{-1},\\ V=\left[v_{i1},\ldots ,v_{iD_{i}}\right]\in {\mathbb{R}}^{P\times D_{i}}, \end{array}$$

**v**_*i*1_,…, **v**_*iD*_*i*__ are vectors spanning ${\hat{S}}_{i}$ described as

(16) $$\begin{array}{c} \mathrm{Span}\left\{v_{i1},\ldots ,v_{iD_{i}}\right\}={\hat{𝒮}}_{i}, \end{array}$$

where **I**_*P*_ is the *P* × *P* identify matrix and *ζ* is an eigenvalue.

The third subproblem is to optimize **b**_*i*_ while fixing **w**_*i*_ and **h**_*i*_. The cost of (9) can be reduced to

(17) $$\begin{array}{c} J_{3}\left(b_{i}\;|\;w_{i},h_{i}\right)\;=\frac{g_{1}\left(b_{i}\right)}{g_{1}\left(b_{i}\right)+g_{2}\left(b_{i}\right)}+\frac{\epsilon }{K}||b_{i}||. \end{array}$$

Then,

(18) gd(b)=EX∈𝒞d[1||b||∑n=1K(bnx~n−1||b||∑k=1Kbkx~k)2]=1||b||EX∈𝒞d[bT(ξ+2μx~)+μ],

where ${\tilde{x}}_{n}=w_{i}^{T}A_{n}h_{i}$, $\tilde{x}={\left[{\tilde{x}}_{1},\ldots ,{\tilde{x}}_{K}\right]}^{T}$, $\xi ={\left[{\tilde{x}}_{1}^{2},\ldots ,{\tilde{x}}_{K}^{2}\right]}^{T}$, and $\mu ={||b||}^{-1}b^{T}\tilde{x}$. Because **b**_*i*_ are chosen out of *ℬ*, we calculate the values $J_{3}({\hat{b}}_{i})$ for all candidates in *ℬ* and the optimal **b**_*i*_ can be chosen as the candidates that maximize *J*_3_. This formulates that

(19) $$\begin{array}{c} b_{i}=\underset{b\in ℬ}{\text{arg max}}\;J_{3}\left(b\right). \end{array}$$

The procedure to design the spatial weights, the temporal filters, and the time windows is summarized in Algorithm 1 as a pseudocode.

### 2.4. Feature Vector Definition

 With designed **w**_*i*_, **h**_*i*_, and **b**_*i*_, the feature vector of an EEG signal, **X**, for classification is defined as

(20) y=[αX(w^1(1),h1,b1),…,αX(w^1(2r),h1,b1),…,αX(w^F(1),hF,bF),…,αX(w^F(2r),hF,bF)]T,

where the set of $\left\{{\hat{w}}_{i}^{\left(1\right)},\ldots ,{\hat{w}}_{i}^{\left(2r\right)}\right\}$ is the CSPs corresponding to **h**_*i*_ and **b**_*i*_. ${\hat{w}}_{i}^{\left(m\right)}$, *i* = 1,…, *F*, *m* = 1,…, 2*r*, are decided as follows. By solving (11) with **h**_*i*_ and **b**_*i*_, we obtain *MF* spatial patterns as ${\tilde{w}}_{i}^{\left(m\right)}$ for *i* = 1,…, *F* and *m* = 1,…, *M*, where ${\tilde{w}}_{i}^{\left(m\right)}$ is the unit-length eigenvector corresponding to the *m*th largest eigenvalue of (11). Then, ${\hat{w}}_{i}^{\left(m\right)}$ are defined as ${\hat{w}}_{i}^{\left(j\right)}={\tilde{w}}_{i}^{\left(j\right)}$ and ${\hat{w}}_{i}^{\left(r+j\right)}={\tilde{w}}_{i}^{\left(M-j+1\right)}$ for *i* = 1,…, *F*,  *j* = 1,…, *r*.

## 3. Experimental Analysis of Artificial Signal

**Figure 2:**
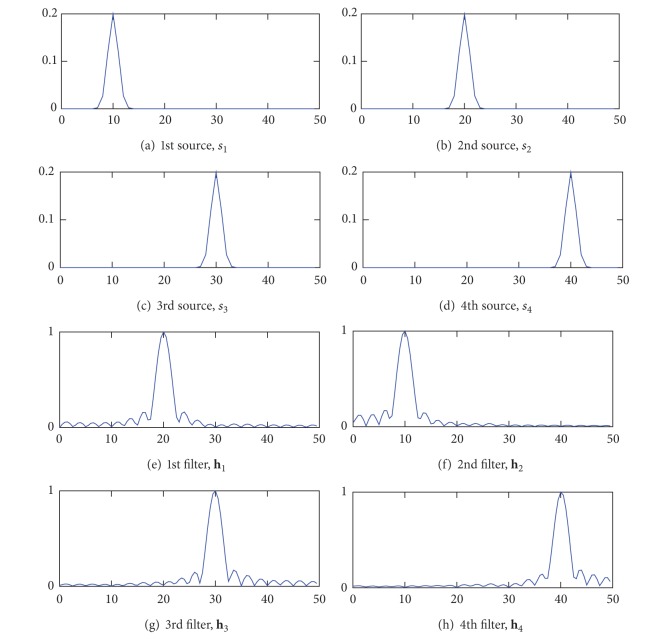
Frequency patterns of the spectra of the source signals ((a)–(d)) and the amplitude characteristics of the designed frequency patterns ((e)–(h)). The horizontal axis represents frequency and its unit is Hz.

We give an analysis of CSTFP by a toy experiment with an artificial signal in this section. We assume a 2-class BMI where the observed EEG signals are modeled by a mixture of narrow-band signals (see Figure 2). In this model, a trial signal belonging to class *d* is given by

(21) $$\begin{array}{c} x\left[n\right]=\overset{N_{s}}{\underset{i=1}{\sum }}s_{i}\left[n\right]a_{i}^{\left(d\right)}+\eta ,\;n=0,\ldots ,N-1, \end{array}$$

where *d* is a class label taking either 1 or 2, **x**[*n*] ∈ ℝ^*M*^ is a vector representing a signal at a discrete time point *n*, *N* is the number of time samples for a trial, *M* is the number of channels, *s*_*i*_[*n*] ∈ ℝ is an *i*th source signal of feature components, *N*_*s*_ is the number of the source signals, **a**_*i*_^(*d*)^ ∈ ℝ^*M*^ is a vector defined as **a**_*i*_^(*d*)^ = [*a*_*i*1_^(*d*)^,…, *a*_*iM*_^(*d*)^]^*T*^, *a*_*im*_^(*d*)^ ∈ ℝ is the amplitude of *s*_*i*_[*n*] in the *m*th channel for class *d*, and **η** ∈ ℝ^*M*^ is a stochastic noise. The source signals, *s*_*i*_[*n*], are generated as

(22) $$\begin{array}{c} s_{i}\left[n\right]=t_{i}\left[n\right]ℜ\left[\overset{N-1}{\underset{k=0}{\sum }}S_{i}\left[k\right]e^{j\theta }e^{j(2\pi k/N)n}\right], \end{array}$$

where *S*_*i*_[*k*] ∈ ℝ represents a discrete spectrum, *t*_*i*_[*n*] represents the time window that decides the period when the *i*th source signal is generated, *θ* ∈ ℝ is a stochastic phase of the source signals, and the operator denoted by *ℜ* takes a real part of a complex number.

In particular, 200 artificial signals that we used in the experiment were generated with the conditions shown in Table 1 where *𝒩*(*m*, *σ* ^2^) is a Gaussian distribution with a mean, *m*, and a variance, *σ* ^2^, and *𝒰*(*a*, *b*) is a uniform distribution whose minimum and maximum values are denoted by *a* and *b*, respectively. 

We applied the CSTFP method to the artificial signals as follows. The class label represented by *c* in (8) was set to 1. The number of the sets of the parameters, *F*, was set to 4. The order of the temporal filters was set to 41, yielding that *P* = 41; the length of a filtered signal, *K*, is 60. For the given candidates for the time windows, we define the following set. First, we define ten *K*-dimensional vectors as

(23) $$\begin{array}{c} d_{j}={\left[\underset{D(j-1)}{\underset{︸}{0,\ldots ,0}},\underset{D}{\underset{︸}{1,\ldots ,1}},0,\ldots ,0\right]}^{T},\;j=1,\ldots ,10, \end{array}$$

where *D* = 6. Then, we use all combinations of {**d**_*j*_}_*j*=1_^10^ represented by

(24) $$\begin{array}{c} {\hat{b}}_{l}=p_{1}d_{1}+\cdots +p_{10}d_{10} \end{array}$$

and *p*_*j*_ ∈ {0,1}, *j* = 1,…, 10, as the given candidate set. Therefore, the number of the candidates, *L*, was 1023. Moreover, for ${\hat{𝒮}}_{i}$ that determines the search space for **h**_*i*_, we used ${\hat{𝒮}}_{i}=\mathrm{Span}\{h_{1},\ldots ,h_{i-1},h_{i-1}^{\prime },\ldots ,h_{i-1}^{\prime }\}$ [15], where each element of **h**_*j*_′ is defined as [**h**_*j*_′]_*k*_ = [**h**_*j*_]_((*k*+1)mod⁡*P*)_, *k* = 1,…, *P*, and the operator denoted by *a*mod⁡*b* takes a residue of dividing *a* by *b*. In addition, the regularization parameter, *ϵ*, was set to 0.05.

**Figure 3:**
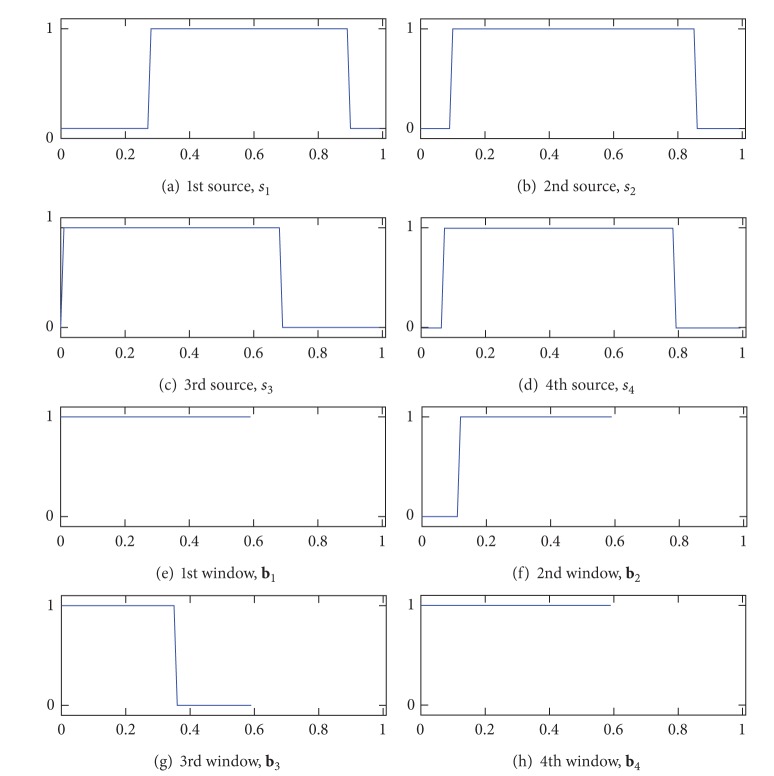
Time patterns as the time windows of the source signals ((a)–(d)) and the designed time windows ((e)–(h)). The horizontal axis represents time and its unit is the second.

**Figure 4:**
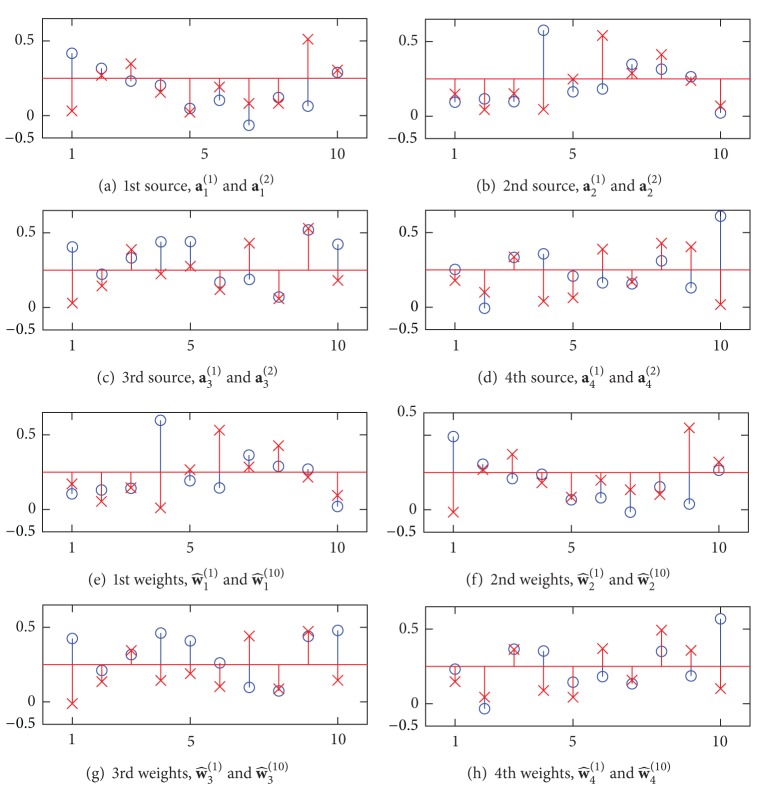
Spatial patterns as the spatial amplitudes of the source signals ((a)–(d)) and the designed spatial weights ((e)–(h)). The amplitudes plotted by circles are **a**_*i*_^(1)^ and the amplitudes plotted by cross are **a**_*i*_^(2)^ for *i* = 1,…, 4. The weights plotted by circles are **w**_*i*_^(1)^ and the weights plotted by cross are **w**_*i*_^(10)^ for *i* = 1,…, 4. The horizontal axis represents the channel number.

The optimization resulted in Figures 2(e)–2(h) for the amplitude characteristics of the FIR filters, Figures 3(e)–3(h) for the time windows, and Figures 4(e)–4(h) for the normalized spatial weights. The centers of passbands of the filters shown in Figures 2(e)–2(h) coincide with the centers of the source signals shown in Figures 2(a)–2(d). Moreover, in the spectrum of the source and the amplitude characteristic of the designed FIR filter that have similar center frequencies, the spatial amplitude corresponding to the source and the spatial weight vector corresponding to the FIR filter are similar to each other. For instance, the correlation coefficient between **a**_1_^(1)^ shown in Figure 4(a) with circles and **w**_2_^(1)^ shown in Figure 4(f) with circles is 0.971.

The results also suggest that the time windows designed by the CSTFP method can remove samples observed in the periods of time that do not contain the source signals. For instance, hence, the source signal, *s*_1_[*n*], is not observed in the first 25 samples according to Figure 3(a), the time window for extracting *s*_1_[*n*] is expected to remove the first 25 samples. Because |*S*_1_[*k*]| and the amplitude characteristics shown in Figure 2(f) have similar center frequency, we can decide that the time window for extracting *s*_1_[*n*] is the time window shown in Figure 3(f). Although the designed time windows do not coincide with *t*_*i*_[*n*] because **b** is applied to an FIR-filtered signal that is shorter than that of the original one, the time window removes samples in the first 10 samples, as we expected. Moreover, we can decide that the time window shown in Figure 3(c) is for extracting *s*_3_[*n*] due to the same reason. As the observed signals do not have *s*_3_[*n*] in the last 25 samples (see Figure 3(c)), the time window shown in Figure 3(c) removes the samples in the last 20 samples.

## 4. Experiment of EEG Signal Classification

 A comprehensive comparative study was performed to illustrate the ability of the CSTFP method to produce more accurate classification of EEG signals during motor imagery over several conventional methods (CSP [10], common sparse spectral spatial patterns (CSSSP) [12], filter bank CSP (FBCSP) [17], and discriminative filter bank CSP (DFBCSP) [15]).

### 4.1. Data Description

 We used dataset IVa from BCI competition III [22], which was provided by Fraunhofer FIRST (Intelligent Data Analysis Group) and Campus Benjamin Franklin of the Charité - University Medicine Berlin (Department of Neurology, Neurophysics Group) [23] and dataset 1 from BCI competition IV, which was provided by Berlin Institute of Technology (Machine Learning Laboratory), Fraunhofer FIRST (Intelligent Data Analysis Group), and Campus Benjamin Franklin of the Charité - University Medicine Berlin (Department of Neurology, Neurophysics Group) [24]. The condition for each dataset is shown in Table 2. They have two classes of motor imagery. The signals in the provided datasets were recorded with the sampling rate of 1000 Hz.

We furthermore applied to this dataset a Butterworth low-pass filter whose cutoff frequency is 50 Hz and the filter order is 4, and downsampled to 100 Hz.

### 4.2. Result

For the experiments, as a sample for each trial, we used a signal observed in the period from *T*_1_ to *T*_2_ [sec] after the cue that directs the subject to perform the task. In the experiments, *T*_1_ was tuned by a method we mention later. *T*_2_ was set to 3.5 and 4 seconds for BCI competition III dataset IVa and IV dataset 1, respectively.

In order to compare the classification abilities for the methods, we obtained the classification accuracy rates by 5 × 5 cross-validation (CV). In each classification in the CV, we separated learning samples for selecting the parameters of the feature extraction and a linear discriminant analysis (LDA) classifier and test samples for obtaining classification accuracy rates.

For the methods to be compared (CSP, CSP-Exh, CSSSP, FBCSP, DFBCSP, and CSTFP), the parameters for the feature extraction were obtained as follows.

(i) CSP: the parameters determined in this method are spatial weights. Before obtaining the spatial weights by the CSP method, we applied the Butterworth bandpass filter with the passband of 7–30 Hz. In the CSP method, we minimized the variance cost of the right hand class in (1). The eigenvectors corresponding to the *r* largest and *r* smallest eigenvalues of the eigenvalue problem (2) were given as the spatial weights.
(ii) CSP-Exh: the parameters determined in this method are spatial weights and a passband of the Butterworth filter. The passband of the Butterworth filters was tuned as *f*_*l*_ − *f*_*u*_ Hz by an exhaustive search by the CSP method and the learning samples. After the filtering with the passband, the spatial weights were given by the same manner as that of the CSP method.
(iii) CSSSP: the parameters determined in this method are spatial weights and a bandpass filter. The bandpass filter between 7 and 30 Hz was applied as preprocessing [12]. CSSSP was applied with regularization parameters, *C*, and the parameter for the number of the spatial weights, *r*. The order of the filter was fixed to 16 [12].
(iv) FBCSP: the parameters determined in this method are *r* bandpass filters out of a filter bank and associated spatial weights. FBCSP was applied with the mutual information based best individual feature and a naïve Bayesian Parzen window (NBPW) classifier [17]. The filter bank comprising 9 bandpass filters covering 4–40 Hz was used. All filters were Chebyshev type II filters with a bandwidth of 4 Hz each. In FBCSP, the number of the spatial weights, *N*_*M*_, in each band was set to 8. These parameters were decided by referring to [17].
(v) DFBCSP: the parameters determined in this method are *F* FIR filters and *r* spatial weights associated to each FIR filter. DFBCSP was applied with the FIR filter order of 41 as done in [15]. In optimization, we stopped iteration when error of the cost function between successive iterations becomes under 10^−5^.
(vi) CSTFP: the parameters determined in this method are *F* FIR filters, the corresponding *F* time windows, and *r* spatial weights associated with each FIR filter. We fixed *T*_1_ = 0 to observe the behavior of the resulting time window. CSTFP is applied with the FIR filter order, 41 [15], and the following candidate set for the time windows. The candidate set for the time windows, *ℬ*, consists of the vectors defined as
  (25) $$\begin{array}{c} {\hat{b}}_{l}={\left[\underset{D}{\underset{︸}{0,\ldots ,0}},\underset{O}{\underset{︸}{1,\ldots ,1}},0,\ldots ,0\right]}^{T}, \end{array}$$
  where we choose *D* and *O* out of a set {0,5, 10,…, *K*} such that *O* > 50 and *D* + *O* ≤ *K*. The regularization parameter, *ϵ*, was set to 0.1. In alternating optimization, we initialize **h**_*i*_ as a random vector which is orthonormalized from **v**_1_,…, **v**_*D*_*i*__ and **b**_*i*_ as a vector all the elements of which are one. We stopped iteration when error of the cost function between successive iterations becomes under 10^−5^.

For the obscure parameters such as *r* in the above list, we furthermore tuned them by 5 × 5 CV using the learning samples as done in [25]. We conducted the nested CV [25] with all of the combinations of these parameters and obtained the classification accuracy rates. We adopted the combination that performed the highest rates as the parameters. The parameters tuned by the nested CV in the learning data and the candidates for them are summarized in Table 3.

After we obtained the feature vectors extracted by the filters, spatial weights, and the time windows that are designed by each listed method, we calculated the logarithm of the feature vectors. Then, the LDA for the learning samples was used to obtain a projector onto the 1-dimensional space. The threshold for classification was determined as the middle point of two class averages over the projected learning samples. The feature vectors from the test samples were classified by the projection and the threshold and we obtained the classification accuracy rate in each CV. In Tables 4 and 5, CSTFP results in the highest accuracy rate in the average over whole subjects. 

**Figure 5:**
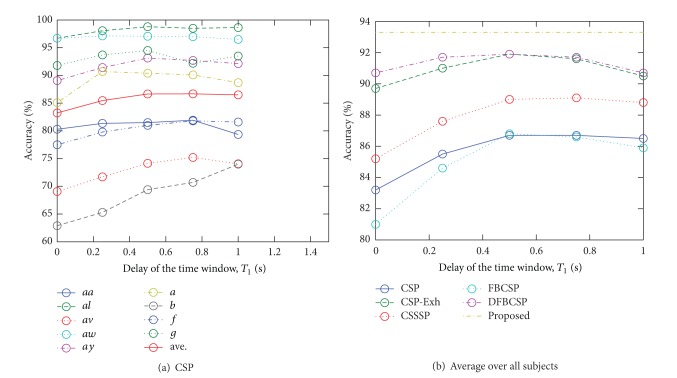
The variation of the accuracy rates by various *T*_1_.

**Figure 6:**
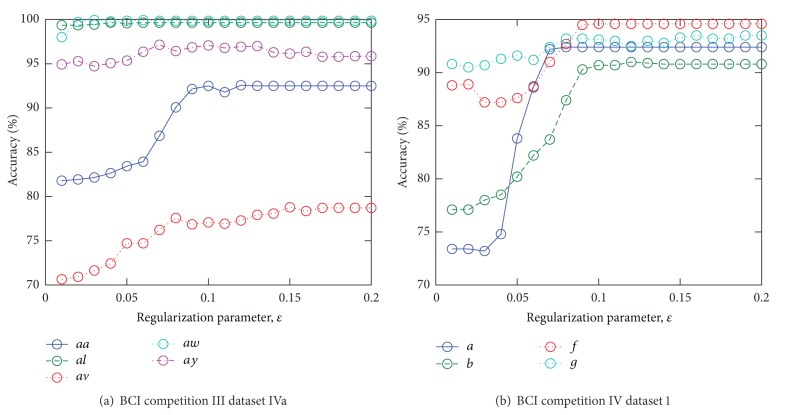
The variation of the accuracy rates by various regularization parameters, *ϵ*.

Moreover, we conducted the classification experiments, in which the parameters shown in Table 3 were fixed to show the effects of the changes of the parameters on the classification accuracy rates.  Figure 5 shows the accuracy rates of CSP and the rates of each method averaged over all subjects with each *T*_1_. For each subject, the parameters shown in Table 3 except for *T*_1_ were fixed to the combinations of the parameters that performed the highest accuracy rates with each *T*_1_. In Figure 5(a), *T*_1_ that perform the highest accuracy rates are different among the subjects. Moreover, Figure 5(b) shows that the accuracy rates highly depend on *T*_1_ in the conventional methods.  Figure 6 shows the variation of the accuracy rates by the various regularization parameters, *ϵ*, in CSTFP. For each subject, the parameters, *r* and *F*, were fixed to the combinations of the parameters that performed the highest accuracy rates with each *ϵ*. CSTFP performs higher accuracy as *ϵ* goes higher than 0.1.

**Figure 7:**
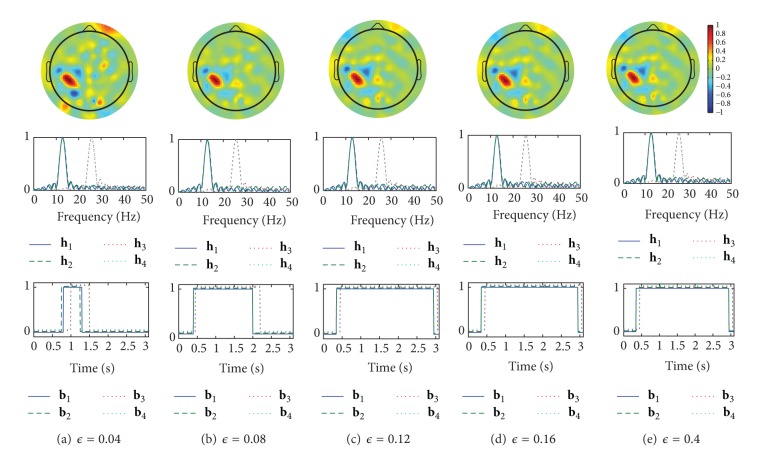
Examples of spatio-time-frequency patterns as the spatial weights, ${\hat{w}}_{1}^{\left(1\right)}$ (top), the amplitude response of the filter, **h**_1_,…, **h**_4_ (center), and the time windows, **b**_1_,…, **b**_4_ (bottom), designed by the CSTFP method with various *ϵ* in the result of subject *aa*. The vertical axis represents normalized amplitude.

**Figure 8:**
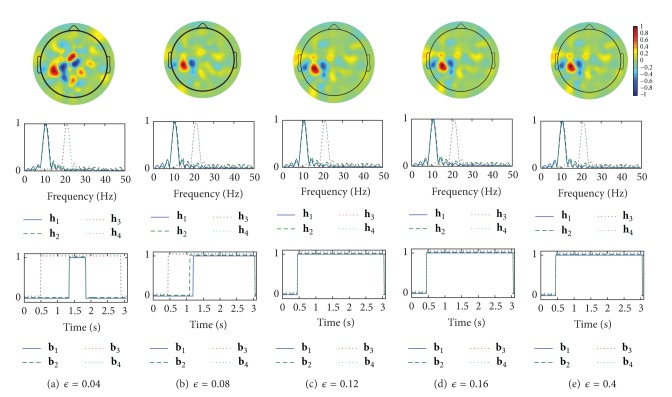
Examples of spatio-time-frequency patterns as the spatial weights, ${\hat{w}}_{1}^{\left(1\right)}$ (top), the amplitude response of the filter, **h**_1_,…, **h**_4_ (center), and the time windows, **b**_1_,…, **b**_4_ (bottom), designed by the CSTFP method with various *ϵ* in the result of subject *av*. The vertical axis represents normalized amplitude.

We show examples of the spatial patterns, the frequency patterns (the amplitude characteristics of the FIR filters), and the time patterns designed by CSTFP in Figures 7 and 8. As we can observe in Figures 6, 7(a), and 8(a), the short time windows caused by small *ϵ* result in poor classification accuracy rates. All of the time windows shown in Figures 7 and 8 remove the samples observed within a few hundreds of milliseconds after the cue. The results suggest that the brain activities related to the task cannot be observed just after the cue. In Figures 7(c)–7(e) and 8(c)–8(e), the time windows do not significantly change in around 0.1–0.4 of *ϵ*. This result of the relations between *ϵ* and the time windows corresponds with the result shown in Figure 6 in which the classification accuracy rates do not strongly depend on the regularization parameter, *ϵ*, by more than 0.12. Moreover, the FIR filters do not highly differ even with various *ϵ*. However, the spatial weights with the small windows (Figures 7(a) and 8(a)) differ from the other weights with the longer windows. Furthermore, there are slight differences in the time patterns and the frequency patterns between subject *aa* and subject *av*. 

## 5. Conclusion

 We have proposed a novel method called CSTFP for the classification of EEG signals during motor imagery. Our objective is to design the time windows that are adopted in signal processing for a cue-based BMI. Incorporating the idea into DFBCSP has allowed us to simultaneously design the parameters for the time windows, the spatial weights, and the FIR filters. These parameters are optimized in a single criterion based on the CSP method. We have shown the optimization procedure for the problem of the CSTFP method that is conducted by sequentially and alternatively solving the subproblems into which the original problem is divided. Through the experiments of the artificial signals and actual EEG signals, we have shown the performance of CSTFP. In the experiment, we have demonstrated that CSTFP achieves high classification accuracy rate. Our experimental results also suggest that the CSTFP method can find the frequency bands and the time periods in which brain activities associated with a mental task can be observed.

Methods for finding time intervals in which the feature components associated to some brain activities are observed are needed for accurate classification in an asynchronous BCI/BMI [26]. We would like to develop such methods by applying the proposed CSTFP algorithm to our future works.

## Figures and Tables

**Algorithm 1:**
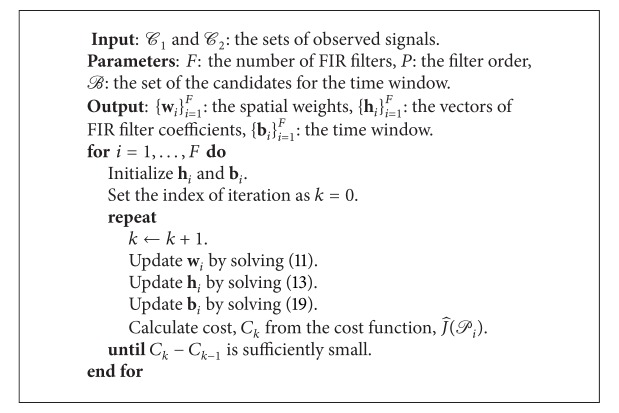
Design of the FIR filters, the spatial weights, and the time windows.

**Table 1:** The conditions for generating the artificial signals.

Parameter	Value or distributions
Number of channel, *M*	10
Number of samples, *N*	100
Number of trials for each class	100
Sampling frequency	100 Hz
Number of sources, *N*_*s*_	4
Spectra of sources, |*S*_*i*_[*k*]|	Figures 2(a)–2(d)
Stochastic phase, *θ*	*𝒰*(0,2*π*)
Time windows, *t*_*i*_[*n*]	Figures 3(a)–3(d)
Amplitudes, **a**_*i*_^(*d*)^	Figures 4(a)–4(d)
Stochastic noise, [**η**]_*m*_	*𝒩*(0,0.1)

**Table 2:** Description of the datasets.

	Dataset IVa	Dataset 1
Classes	Right hand and right foot	2 tasks from foot, left hand, and right hand
Subject labels	*aa*, *al*, *av*, *aw*, *ay*	*a*, *b*, *f*, *g*
Number of channels	118	59
Signal length	3.5 secs	4 secs
Sampling rate	100 Hz	100 Hz
Number of the trials per class	140	100

**Table 3:** The parameters decided by the nested CV in the learning data in the classification experiments.

Method	Parameters and candidates
CSP	*T*_1_ ∈ {0,0.25,0.5,0.75}
	*r* ∈ {1,2,…, 10}

CSP-Exh	*T*_1_ ∈ {0,0.25,0.5,0.75}
	*r* ∈ {1,2,…, 10}
	*f*_*l*_ ∈ {1,2,…, 48}
	*f*_*u*_ ∈ {*f*_*l*_ + 1, *f*_*l*_ + 2,…, 49}

CSSSP	*T*_1_ ∈ {0,0.25,0.5,0.75}
	*r* ∈ {1,2,…, 10}
	*C* ∈ {0,0.01,0.1,0.2,0.5,1, 2,5}

FBCSP	*T*_1_ ∈ {0,0.25,0.5,0.75}
	*r* ∈ {1,2,…, 10}

DFBCSP	*T*_1_ ∈ {0,0.25,0.5,0.75}
	*r* ∈ {1,2,…, 10}
	*F* ∈ {1,2,…, 5}

CSTFP	*r* ∈ {1,2,…, 10}
	*F* ∈ {1,2,…, 5}

**Table 4:** Classification accuracy [%] given by 5 × 5 CV in dataset IVa from BCI competition III. The figure with ± represents the standard deviation (S.D.) over CV.

Method	Subject					Average
	* aa *	* al *	* av *	* aw *	* ay *	
CSP	79.9 ± 4.6	98.4 ± 1.6	71.9 ± 3.5	96.8 ± 2.6	92.5 ± 3.1	87.9
CSP-Exh	90.8 ± 3.6	99.1 ± 1.4	74.4 ± 5.5	99.1 ± 1.4	93.0 ± 4.2	91.3
CSSSP	91.5 ± 3.7	99.1 ± 1.6	71.1 ± 6.9	98.8 ± 1.4	93.1 ± 3.5	90.7
FBCSP	91.3 ± 3.4	99.3 ± 1.5	70.7 ± 7.4	98.2 ± 1.6	88.4 ± 4.8	89.6
DFBCSP	91.9 ± 2.6	98.9 ± 1.5	74.8 ± 4.0	98.9 ± 1.6	96.0 ± 3.1	92.1
CSTFP	92.6 ± 2.1	98.9 ± 1.5	75.4 ± 5.3	99.0 ± 1.3	96.0 ± 2.4	** 92.5**

**Table 5:** Classification accuracy [%] given by 5 × 5 CV in dataset 1 from BCI competition IV. The figure with ± represents the standard deviation (S.D.) over CV.

Method	Subject				Average
	* a *	* b *	* f *	* g *	
CSP	89.6 ± 5.1	68.8 ± 6.7	78.1 ± 6.5	93.9 ± 3.4	82.6
CSP-Exh	92.3 ± 2.5	85.3 ± 6.9	78.1 ± 6.5	93.9 ± 3.6	90.2
CSSSP	89.8 ± 5.5	70.4 ± 9.5	85.3 ± 5.3	93.4 ± 4.1	84.7
FBCSP	87.3 ± 8.6	63.9 ± 14.1	78.6 ± 6.9	94.1 ± 4.1	81.0
DFBCSP	89.7 ± 5.0	85.6 ± 6.9	92.5 ± 4.4	93.7 ± 4.3	90.4
CSTFP	91.4 ± 4.0	90.6 ± 7.2	93.3 ± 3.6	93.5 ± 4.1	** 92.2**
